# *Psephellus vanensis* (Asteraceae), a new species from east Turkey

**DOI:** 10.3897/phytokeys.48.8870

**Published:** 2015-04-02

**Authors:** Bekir Dogan, Lütfi Behçet, Ahmet Duran, Davut Avlamaz

**Affiliations:** 1 Necmettin Erbakan University, A. K. Education Faculty, Department of Science Education, TR-42090, Meram-Konya, Turkey; 2 Bingöl University, Faculty of Science and Arts, Department of Biology, 12000, Bingöl, Turkey; 3 Selçuk University, Faculty of Science, Department of Biology, 42075 Selçuklu–Konya, Turkey

**Keywords:** Anatolia, Compositae, taxonomy

## Abstract

A new species, *Psephellus vanensis* A.Duran, Behçet & B.Dogan (Asteraceae) from Anatolia, Turkey, is described and illustrated. The species grows on the serpentine stony field of the village of Çaldıran in the district of Başkale (Van province) in eastern Anatolia. It is morphologically similar to *Psephellus pyrrhoblepharus* (Boiss.) Wagenitz. Diagnostic characters are discussed, and a key to the most similar species is provided. Ecology, conservation status and notes on biogeography of the species are also presented. In addition, the geographical distribution of the new species and other related species in Turkey is mapped.

## Introduction

The genus *Psephellus* Cass. embraces 75–80 species. Its distribution is centered in east Anatolia, the Caucasus and northwest Iran; only few species occur outside this area (Wagenitz and Hellwig 2000).

In Wagenitz and Hellwig (2000), 12 sections that had been included in the genus *Centaurea* were transferred to the genus *Psephellus*, namely Psephellus sect. Psephelloideae (Boiss.) Wagenitz & Hellwig, Psephellus sect. Psephellus (Cass.) Wagenitz & Hellwig, Psephellus sect. Hyalinella (Tzelev) Wagenitz & Hellwig, Psephellus sect. Aetheopappus (Cass.) Wagenitz & Hellwig, Psephellus sect. Odontolophus (Cass.) Wagenitz & Hellwig, Psephellus sect. Xanthopsis (DC.) Wagenitz & Hellwig, Psephellus sect. Amblyopogon (DC.) Wagenitz & Hellwig, Psephellus sect. Heterolophus (Cass.) Wagenitz & Hellwig, Psephellus sect. Czerniakovskya (Czerep.) Wagenitz & Hellwig, Psephellus sect. Odontolophoideae (Tzvelev) Wagenitz & Hellwig, Psephellus sect. Uralepis (DC.) Wagenitz & Hellwig and Psephellus sect. Sosnovskya (Takht.) Wagenitz & Hellwig. New combinations under the genus *Psephellus* were provided for these sections and 35 species, especially from Turkey and Iran. Some of these species occur only in Turkey.

In Turkey, *Psephellus* is represented by 31 species including some recently described species. After Wagenitz and Hellwig (2000), *Psephellus turcicus* A.Duran & E.Hamzaoglu, *Psephellus recepii* Wagenitz & Kandemir, *Psephellus erzincanii* Wagenitz & Kandemir, *Psephellus coruhensis* A.Duran & M.Öztürk, *Psephellus yusufeliensis* O. Tugay & Uysal were described (Duran and Hamzaoğlu 2005, Wagenitz and Kandemir 2008, Duran et al. 2009, Tugay et al. 2009). *Psephellus yusufeliensis* was reduced to a synonym of *Psephellus coruhensis* (Duran et al. 2014).

During a field trip, some specimens of the genus *Psephellus* were collected in easthern Anatolia, in the Van province. After examining carefully the specimens and the descriptions of *Psephellus* species in Wagenitz (1975), Dostal (1976), Davis et al. (1988), Wagenitz et al. (1998), Wagenitz and Hellwig (2000), Güner (2000), Duran and Hamzaoğlu (2005), Wagenitz and Kandemir (2008), Duran et al. (2009), as well as comparing with specimens in the Herbaria KNYA, ANK, GAZI, GOET, HUB, E, K and BM, it was determined that our specimens represent a species new to science. In this paper, this new species of *Psephellus* is described and illustrated.

In the description below, each numerical value is the average of ten measurements from different specimens. Our specimens of *Psephellus vanensis* sp. nov. were examined and compared with specimens of the related species *Psephellus pyrrhoblepharus* and *Psephellus gilanicus* collected in Turkey. With the new species described here, the total number of taxa in the genus *Psephellus* has risen to 32 in Turkey.

## Taxonomic treatment

### Psephellus vanensis

Taxon classificationPlantaeAsteralesAsteraceae

A. Duran, Behçet & B. Doğan sp. nov.

urn:lsid:ipni.org:names:77146129-1

Figs 1 , 2 , 3

#### Diagnosis.

*Psephellus vanensis* differs from *Psephellus pyrrhoblepharus* in its stem 13–20 cm tall and tomentose (vs. (20–)30–50 cm, floccose-tomentose), basal leaves usually undivided and elliptic to lanceolate, rarely lyrate with 2–3 pairs of lateral segments (vs. lyrate with very large broadly lanceolate terminal segment and 1–2 pairs of small lateral segment), involucre 11–17 × 11–14 mm, bowl-shaped (vs. 20–25 × 15–25 mm, ovoid to nearly globose), achenes 4–5 mm long (vs. 6–7 mm), pappus 5–6 mm long (vs. 4–7 mm long), inner row of scales 1–2 mm long (vs. 3–4 mm long).

#### Type.

**Turkey. Van:** Başkale, Çaldıran village, steppe fields, 2000–2050 m a.s.l., 17 Jun 2009, *Behçet & D. Avlamaz 1603* (holotype: KNYA, isotypes: GAZI, ANK, HUB, Bingöl Univ. Herb.).

#### Description.

Perennial herb with a woody rootstock. Stem erect, striate, densely tomentose, 13–20 cm tall, 1.3–2 mm in diameter at base, simple, upper parts of stems leafless. Leaves concoloured, green, densely tomentose; basal leaves usually undivided and elliptic to lanceolate, 3–7 × 0.6–1.2 cm (including petiole), rarely lyrate with 2–3 pairs of lateral segments; cauline and upper cauline leaves undivided and lanceolate, partly decreasing in size towards capitula, 1–2.5 × 0.2–0.9 cm. Capitula solitary, 18–28 × 11–15 mm (including flowers). Involucre 11–16 × 11–14 mm, bowl-shaped. Phyllaries nearly imbricate, glabrous; appendages conspicuous, large, concealing most of the basal part of phyllaries, scarious, pale-brownish, with distinct cilia, cilia 1–2 mm long, 8–10 cilia on each side. Corolla pink-violet. Marginal florets slightly longer than central florets, radiant, 12–13 mm long, without staminode, with 5 narrowly linear-lanceolate lobes 3–4 mm long; central flowers radiant, 10–11 mm long, without staminode, with 5 lobes 2–3 mm long. Achenes 4–5 mm, straw-colored to brownish, smooth, glabrous; pappus 5–6 mm, inner row of scales 1–2 mm, scabrous. Flowers in June-July and fruits in July–August.

**Figure 1.**
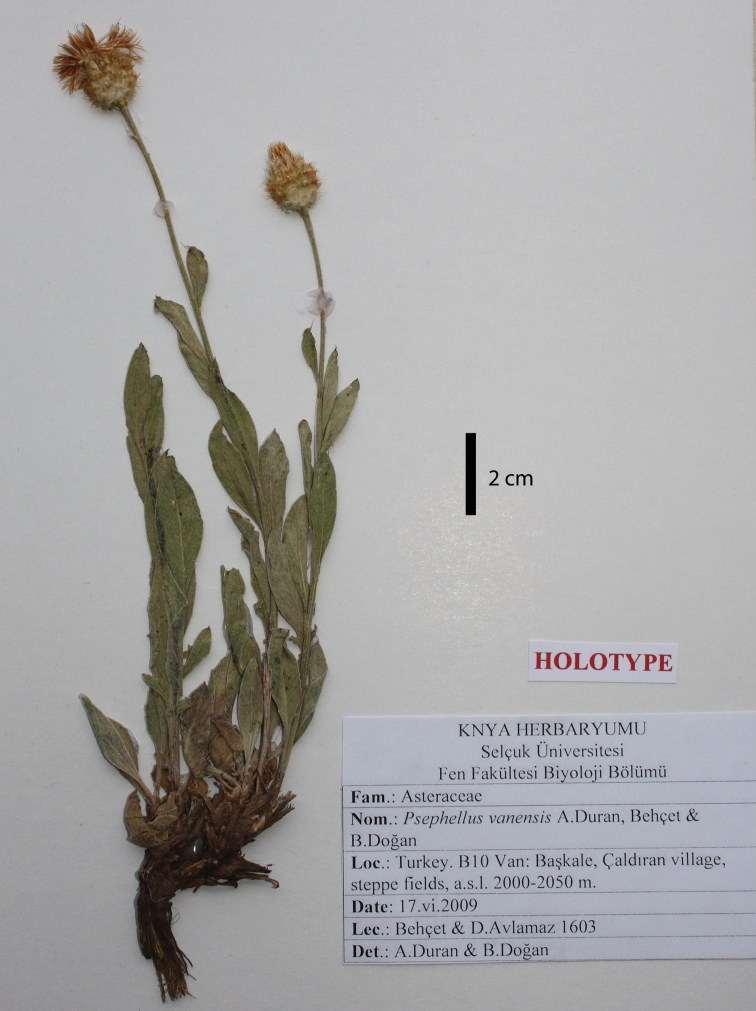
Holotype of *Psephellus vanensis* A.Duran, Behçet & B.Doğan.

**Figure 2.**
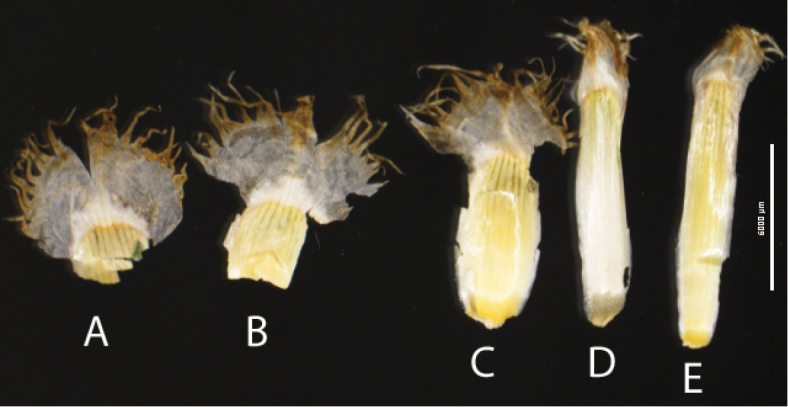
Phyllaries of *Psephellus vanensis*. **A, B** Outer phyllaries **C** Median phyllaries **D, E** Inner phyllaries. Scale bar: 6000 µm.

**Figure 3.**
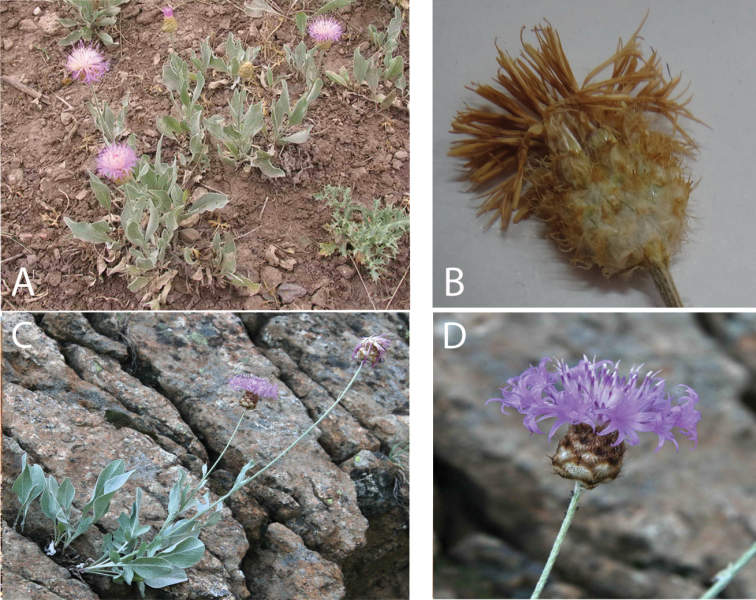
*Psephellus vanensis*. **A** Habit **B** Capitulum at anthesis. *Psephellus pyrrhoblepharus*. **C** Habit **D** Capitulum at anthesis.

#### Ecology.

The new species occurs on steppe fields, whereas *Psephellus pyrrhoblepharus* is found on rocks and slopes. *Psephellus vanensis* grows in plant communities with *Asyneuma pulchellum* (Fischer & C.A.Mey.) Bornm., *Campanula conferta* DC., *Tanacetum kotschyi* (Boiss.) Grierson, *Bromus danthoniae* Trin., *Bromus tomentellus* Boiss., *Eryngium billardieri* Delar., *Helichrysum plicatum* DC., Thymus kotschyanus Boiss. & Hohen. var. kotschyanus, *Ziziphora clinopodioides* Lam., *Achillea vermicularis* Trin., Gundelia tournefortii L. var. tournefortii, *Erysimum echinellum* Hand.-Mazz., *Iris paradoxa* Steven, Dactylis glomerata L. subsp. glomerata, *Stipa pontica* P.Smirnov, *Prangos pabularia* Lindley and *Dianthus orientalis* Adams.

#### Distribution and conservation status.

**Figure 4.**
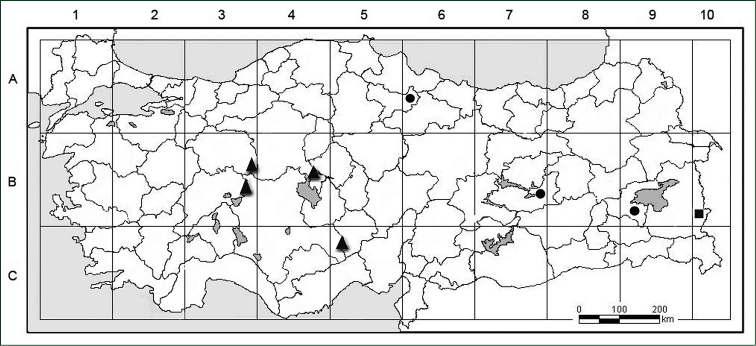
Distribution maps of *Psephellus vanensis* (■), *Psephellus pyrrhoblepharus* (●) and *Psephellus bornmuelleri* (▲) in Turkey.

*Psephellus vanensis* is endemic to east Anatolia, where it seems to be very local. It belongs to the Irano-Turanian element (Fig. 4). The species is known only from type gatherings and from an area of approximately 0,006 km^2^ (criterion B1). Because of overgrazing, the habitat of this species is under threat, and this situation leads to potential reduction in the number of individuals (criterion A). The population is in a poor condition, and the number of individuals is estimated to approximately 120–125 (criterion C2). Therefore the species should be regarded as Critically Endangered (IUCN 2014).

#### Key to the related *Psephellus* species

**Table:** 

1	Stem taller than 20 cm, appendages straw-coloured, achenes 6–7 mm long	**2**
–	Stem shorter than 20 cm, appendages pale brownish, achenes 4–5 mm long	***Psephellus vanensis***
2	Cauline leaves pinnatilobate or undivided, appendages with ciliae 3–5 mm long, pappus 4–7 mm long	***Psephellus pyrrhoblepharus***
–	Cauline leaves lanceolate to linear-lanceolate, appendages with ciliae 1–2 mm long, pappus 8–9 mm long	***Psephellus gilanicus***

#### Taxonomic position.

The new species is placed in Psephellus sect. Psephelloidei (Boiss.) Wagenitz & Hellwig according to the involucre and achene characters as determined by Wagenitz (1975), Wagenitz and Hellwig (2000).

#### Affinity.

*Psephellus vanensis* is closely related to *Psephellus pyrrhoblepharus*, which occurs in Central Anatolia and is endemic to Turkey. It mainly differs from *Psephellus pyrrhoblepharus* in its stem 13–20 cm tall and densely tomentose (vs. 30–50 cm, floccose-tomentose), basal leaves usually undivided and elliptic to lanceolate, rarely lyrate with 2–3 pairs of lateral segments (vs. lyrate with very large broadly lanceolate terminal segment and 1–2 pairs of small lateral segments).

*Psephellus vanensis* is also related to *Psephellus gilanica*, which is endemic to Iran (Wagenitz 1980). It mainly differs from *Psephellus gilanica* in its stem 13–20 cm tall (vs. 25–40 cm), tomentose (vs. sparsely floccose-tomentose), basal leaves usually undivided and lanceolate, rarely lyrate with 2–3 pairs of lateral segments (vs. lyrate with 2–3 pairs of lateral segments).

Additional characters of *Psephellus vanensis* and the related species *Psephellus gilanica* and *Psephellus pyrrhoblepharus* are provided in Table 1.

**Table 1.** Diagnostic characters of *Psephellus vanensis*, *Psephellus gilanicus* and *Psephellus pyrrhoblepharus*.

Characters	*Psephellus vanensis*	*Psephellus pyrrhoblepharus*	*Psephellus gilanicus*
Stem	13–20 cm tall, tomentose	30–50 cm tall, floccose-tomentose	25–40 mm tall, sparsely floccose-tomentose
Basal leaves	usually undivided and elliptic to lanceolate, rarely lyrate with 2–3 pairs of lateral segment	lyrate with very large broadly lanceolate terminal segment and 1–2 pairs of small lateral segments	lyrate, 2–3 pairs of lateral segments
Cauline leaves	undivided, lanceolate	pinnatilobate or simple	lanceolate to linear-lanceolate
Involucre	11–17 × 11–14 mm, bowl-shaped	20–25 × 15–25 mm, ovoid to nearly globose	(14–)16–20 × (11–)14–18 mm, subglobose
Appendages	pale-brownish	straw-coloured	straw-coloured
Appendages, cilia	8–10 cilia on each side, 1–2 mm long	7–8 cilia on each side, 3–5 mm long	8–14 cilia on each side, 1–2 mm long
Flowers	pink-violet	rose-purple	pink-purple
Achenes	4–5 mm long	6–7 mm long	6–7 mm long
Pappus	5–6 mm long, inner row of scales 1–2 mm long	4–7 mm long, inner row of scales 3–4 mm long	8–9 mm long, inner row of scales 2–3 mm long

*Psephellus vanensis* also resembles *Psephellus bornmuelleri*, which occurs in Central Anatolia and is endemic in Turkey. It mainly differs from *Psephellus bornmuelleri* in its stem 13–20 cm tall and tomentose (vs. 35–70 cm, sparsely tomentose to glabrescent), basal leaves usually undivided and elliptic to lanceolate, rarely lyrate with 2–3 pairs of lateral segments (vs. pinnatipartite or lyrate, with 4–6 pairs of lateral segments), involucre 11–17 × 11–14 mm, bowl-shaped (vs. 15–20 × 15–25 mm, ovoid to nearly globose), appendages pale brownish (vs. straw-coloured), flowers pink-violet (vs. purple), achenes 4–5 mm long (vs. c. 7 mm long).

#### Phytogeography.

The east Anatolia region is a botanically interesting area, occupying the Irano-Turanian phyto-geographical region. The area is very rich in local endemic plants (Akman et al. 2011; Koçyiğit and Bona 2013). Recently many articles were published on new species from this particular region, notably *Ferula mervynii* M. Sağıroğlu & H. Duman (Sağıroğlu and Duman 2007), *Silene dumanii* Kandemir, G. Ecevit Genç & İ. Genç (Kandemir and Genç 2009), *Jurinea tortumensis* A. Duran & B. Dogan (Dogan et al. 2010), *Campanula hacerae* A. İlçim (İlçim et al. 2011), *Silene gevasica* Hamzaoğlu (Hamzaoğlu et al. 2011), *Allium shirnakiense* L. Behçet & Rüstemoğlu (Behçet and Rüstemoğlu 2012), *Rhabdosciadium urusakii* E. Akalın (Akalın and Akpulat 2012), *Onosma atila-ocakii* O Koyuncu & Yaylacı (Koyuncu et al. 2013) and *Crocus yakarianus* Yıldırım & O. Erol (Yıldırım and Erol 2013).

#### Additional specimens examined.

*Psephellus pyrrhoblepharus*: Turkey, B7 Elazığ: Harput, around the Anguzu Baba Türbesi, 1560 m., 14 Jun 2007, *A. Duran 7464, B. Dogan & M. Öztürk* (KNYA!); A6 Amasya: Akdağ, above Zefe köy, 1700 m., *Tobey 1207* (E, photo!); B9 Bitlis: Kambos Da., above Hürmüz, 1800 m., 31 Jun 1954, *Davis 23403* (E, photo!).

*Psephellus bornmuelleri*: Turkey, C5 Konya: between Ereğli-Niğde, 1400 m, 1904, *W.Siehe* (E, photo!); B3 Eskisehir: c. 15 miles from Polatli to Sivrihisar, 800 m, 12 Jun 1965, chalky fields, *Coode & Jones* 2252 (E, photo!); Ankara: Polatlı, Acıkır vicinity, 840–860 m, 2 Jun 1995, gypsum places, *Aytaç* 6893 & *Adigüzel* (GAZI!); Ankara: Polatlı, Acıkır vicinity, 840–860 m, 22 Jun 1993, *Duman* 4812 & *Aytaç* (GAZI!); Ankara: Polatlı, Acıkır vicinity, 840–860 m, 4 Jun 1991, *Aytaç* 3822 & *Duman* (GAZI!); B4 Ankara, between Şereflikoçhisar-Ankara, 10 km, saline places, 900–950 m, 5 Jun 2002, *Aytaç* 8374 & *M.Ekici* (GAZI!).

*Psephellus gilanica*: Iran, Tehran: prope Shekerabad, 2200 m, *Bornmüller 7266* (B, photo!).

Note: Davis’s grid system was used for the coordinates.

## Supplementary Material

XML Treatment for Psephellus vanensis
